# Supplementary material for: Statistical Power Analysis for Designing Bulk, Single-Cell, and Spatial Transcriptomics Experiments: Review, Tutorial, and Perspectives
Source: Biomolecules. 2023 Jan 24;13(2):221. doi: 10.3390/biom13020221 (PMC9952882; doi:10.3390/biom13020221)
Supplement: Supplementary file 1 [file biomolecules-13-00221-s001.zip › Supplementary Material S1.pdf]

Supporting Information for 'Statistical Power Analysis for Designing Bulk, Single-Cell, and Spatial Transcriptomics Experiments: Review, Tutorial, and Perspectives'

Supplementary Material S1. Tutorial for 'ssizeRNA' R package

Hyeongseon Jeon[1,2,†], Juan Xie[1,2,3,†], Yeseul Jeon[1,4,5,†], Kyeong Joo Jung[6], Arkobrato Gupta[1,2,3], Won Chang[7], Dongjun Chung[1,2,*]

[1] Department of Biomedical Informatics, The Ohio State University, Columbus, OH, U.S.A.
[2] Pelotonia Institute for Immuno-Oncology, The James Comprehensive Cancer Center, The Ohio State University, Columbus, OH 43210, U.S.A.
[3] The Interdisciplinary PhD program in Biostatistics, The Ohio State University, Columbus, Ohio, U.S.A.
[4] Department of Statistics and Data Science, Yonsei University, Seoul, South Korea
[5] Department of Applied Statistics, Yonsei University, Seoul, South Korea
[6] Department of Computer Science and Engineering, The Ohio State University, Columbus, Ohio, U.S.A.
[7] Division of Statistics and Data Science, University of Cincinnati, Cincinnati, Ohio, U.S.A.
[†] These authors contributed equally to this work
[*] Correspondence: chung.911@osu.edu

Step 1: Load packages (ssizeRNA and edgeR)

The following script demonstrates how to install the necessary R/Bioconductor packages for this tutorial. Note that the edgeR Bioconductor package is utilized to estimate dispersion parameters.

```
##  Step 1-1 Install ssizeRNA package
# install.packages("ssizeRNA")

##  Step 1-2 Install other packages in the tutorial if needed.
# install.packages("BiocManager")
# BiocManager::install("edgeR") # cf. https://bioconductor.org/packages/release/bioc/html/edgeR.html
# BiocManager::install("Biobase") # cf. https://bioconductor.org/packages/release/bioc/html/Biobase.html

library(ssizeRNA)

library(edgeR)
```

```
library(Biobase)
```

Step 2: Prepare parameter estimates using comparable data set.

The following script describes obtaining the necessary model parameter estimates to implement the core function called ssizeRNA_vary. The necessary parameters to be estimated are the average read count in the control group (mu) and dispersion parameter estimates (disp). Note that the number of estimated parameters can differ from the required number of target genes (nGenes). Note that it is highly encouraged to replace the example data with similar data of your choice.

```
##  Example data saved in ssizeRNA package:
##  Step 2-1. load hammer dataset (Hammer, P. et al., 2010)

data(hammer.eset)
counts <- exprs(hammer.eset)[, phenoData(hammer.eset)$Time == "2 weeks"]
counts <- counts[rowSums(counts) > 0,]
trt <- hammer.eset$protocol[which(hammer.eset$Time == "2 weeks")]

##  After generating count data with column names of control and treatment, you may est
imate the parameters of mu and disp using the following script.

##  mu: average read count in the control group
##  The following apply function averages the count values for each gene in the control g
roup.


mu <- apply(counts[, trt == "control"], 1, mean)

##  disp: dispersion parameters estimates using the edgeR package with count data.


d <- DGEList(counts)
d <- calcNormFactors(d)
d <- estimateCommonDisp(d)
d <- estimateTagwiseDisp(d)
disp <- d$tagwise.dispersion
```

Step 3: Define additional input variables

Before executing the core function ssizeRNA_vary, you need to determine additional input variables: the number of genes (nGenes), non-DE gene proportion (pi0), proportion of up-regulated genes among all DE genes (up), desired power (power), the false discovery rate to be

controlled (fdr), and the maximum sample size considered (maxN). The fold change (fc) for DE genes is fixed in this script. (One can implement varying fold changes by assigning the fc variable to a function that determines the fold change values.)

```
# Define additional input parameters.

nGenes = 10000; pi0 = 0.8; fdr = 0.05; power = 0.8; up = 0.5; maxN = 35;
##  fixed fold change
fc <- 2

##  fold change function.
# fc <- function(x){exp(rnorm(x, log(2), 0.5*log(2)))}

## If you wish to assume variable fold changes for DE genes, please check the histogram
 using the following script.
# hist(fc(nGenes))
```

Step 4: Implement ssizeRNA_vary function to estimate sample size required to achieve desired power (power) when controlling a FDR level (fdr) for two-sample RNA-seq experiments in which gene-specific means and dispersions are assumed.

The program creates automatically a power function according to the sample size. Using the curve, you may determine the sample size yourself.

```
size <- ssizeRNA_vary(nGenes = nGenes,
                      mu = mu, disp = disp,
                      fc = fc,
                      up = up, fdr = fdr, power = power,
                      maxN = maxN)
```

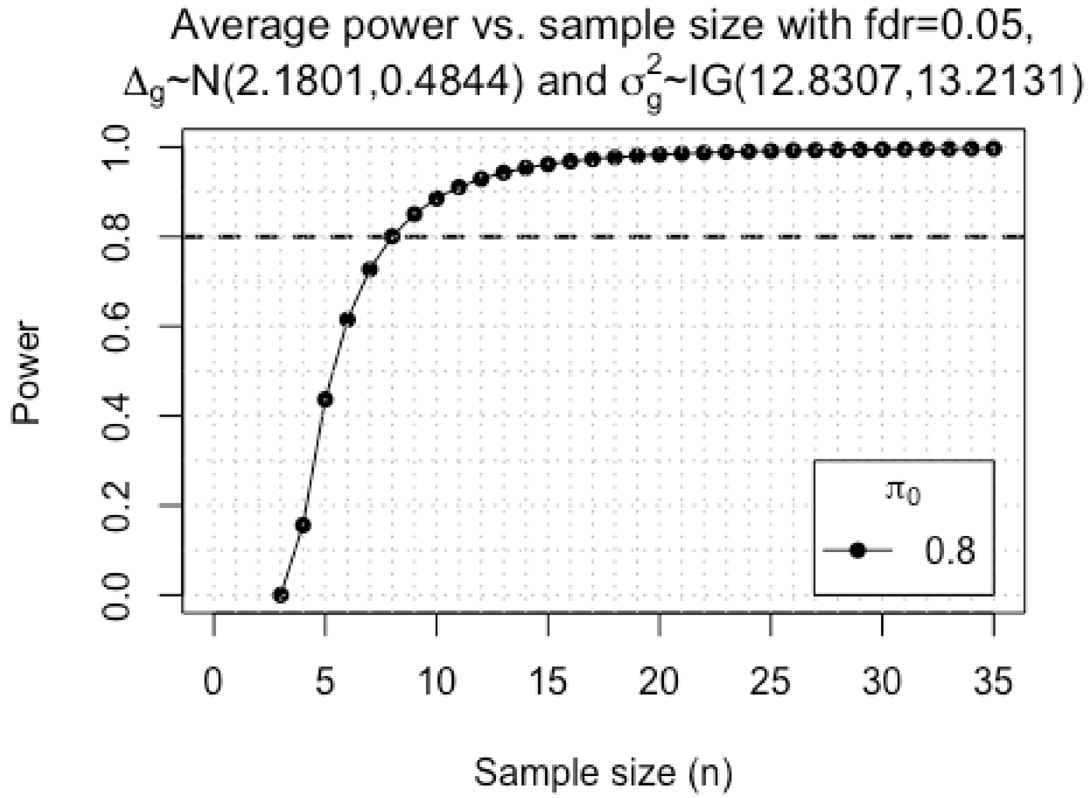

For your convenience, you may determine the sample size using output variables named ssize. The variable power output gives extra information on power for various sample sizes. If the power is not obtained prior to the maximum sample size, you can increase the maxN variable. When power is set to 0.999, for instance, no candidate sample size, from 1 to maxN, can reach the power, hence the maxN variable can be increased.

```
# Output variables:
# ssize: sample sizes (for each treatment) at which desired power is first reached.
size$ssize

##      pi0 ssize     power
## [1,] 0.8     8 0.8006006

# power: power calculations with corresponding sample sizes.
size$power

##        n       0.8
##  [1,]  3 0.0000000
##  [2,]  4 0.1559197
##  [3,]  5 0.4367029
##  [4,]  6 0.6149295
##  [5,]  7 0.7271366
```

```
##  [6,]  8 0.8006006
##  [7,]  9 0.8504863
##  [8,] 10 0.8854384
##  [9,] 11 0.9105686
## [10,] 12 0.9290500
## [11,] 13 0.9429000
## [12,] 14 0.9534637
## [13,] 15 0.9616395
## [14,] 16 0.9680513
## [15,] 17 0.9731421
## [16,] 18 0.9772270
## [17,] 19 0.9805399
## [18,] 20 0.9832498
## [19,] 21 0.9854870
## [20,] 22 0.9873462
## [21,] 23 0.9889036
## [22,] 24 0.9902175
## [23,] 25 0.9913327
## [24,] 26 0.9922842
## [25,] 27 0.9931010
## [26,] 28 0.9938061
## [27,] 29 0.9944173
## [28,] 30 0.9949497
## [29,] 31 0.9954153
## [30,] 32 0.9958245
## [31,] 33 0.9961853
## [32,] 34 0.9965049
## [33,] 35 0.9967885
```
