# Supplementary material for: Statistical Power Analysis for Designing Bulk, Single-Cell, and Spatial Transcriptomics Experiments: Review, Tutorial, and Perspectives
Source: Biomolecules. 2023 Jan 24;13(2):221. doi: 10.3390/biom13020221 (PMC9952882; doi:10.3390/biom13020221)
Supplement: Supplementary file 1 [file biomolecules-13-00221-s001.zip › Supplementary Material S2.pdf]

Supporting Information for 'Statistical Power Analysis for Designing Bulk, Single-Cell, and Spatial Transcriptomics Experiments: Review, Tutorial, and Perspectives'

Supplementary Material S2. Tutorial for 'POWSC' Bioconductor package

Hyeongseon Jeon[1,2,†], Juan Xie[1,2,3,†], Yeseul Jeon[1,4,5,†], Kyeong Joo Jung[6], Arkobrato Gupta[1,2,3], Won Chang[7], Dongjun Chung[1,2,*]

[1] Department of Biomedical Informatics, The Ohio State University, Columbus, OH, U.S.A.
[2] Pelotonia Institute for Immuno-Oncology, The James Comprehensive Cancer Center, The Ohio State University, Columbus, OH 43210, U.S.A.
[3] The Interdisciplinary PhD program in Biostatistics, The Ohio State University, Columbus, Ohio, U.S.A.
[4] Department of Statistics and Data Science, Yonsei University, Seoul, South Korea
[5] Department of Applied Statistics, Yonsei University, Seoul, South Korea
[6] Department of Computer Science and Engineering, The Ohio State University, Columbus, Ohio, U.S.A.
[7] Division of Statistics and Data Science, University of Cincinnati, Cincinnati, Ohio, U.S.A.
[†] These authors contributed equally to this work
[*] Correspondence: chung.911@osu.edu

DEG identification

POWSC can be used to identify genes whose expression changes under different conditions for a particular cell type (within cell type) or to identify biomarkers that distinguish cell types (between cell types). We will provide codes for each scenario.

Template datasets for parameter estimation

POWSC provides two template datasets for parameter estimation: mouse data (GSE29087, with embryonic fibroblast and embryonic stem cells) and human brain data (GSE67835). The mouse data consists of 92 cells with two cell types, and the human brain data consists of 57 cells with four cell types(the original study sequenced 466 cells, POWSC just included partial cells in the package). Both datasets are formatted as SingleCellExperiment objects.

```
suppressMessages(library(POWSC))

## Warning: package 'matrixStats' was built under R version 4.1.3
```

```
## Warning: package 'S4Vectors' was built under R version 4.1.3
```

```r
###  mouse data
#load data
data('es_mef_sce')
# quick view the data
table(es_mef_sce@colData$cellTypes)
```

```
##
##    fibro stemCell
##       44       48
```

```r
# load human brain data
sce
```

```
## class: SingleCellExperiment
## dim: 1000 57
## metadata(0):
## assays(1): counts
## rownames(1000): STIP1 STS ... LINC00311 PNKP
## rowData names(1): geneNames
## colnames(57): GSM1658127 GSM1658128 ... GSM1658182 GSM1658183
## colData names(3): tissueTypes cellTypes Patients
## reducedDimNames(0):
## mainExpName: NULL
## altExpNames(0):
```

```r
# quick view of the data
table(sce@colData$cellTypes)
```

```
##
##       astrocytes           hybrid          neurons oligodendrocytes
##                3                7               35               12
```

```r
table(sce@colData$tissueTypes)
```

```
##
## cortex
##     57
```

```r
table(sce@colData$Patients)
```

```
##
## AB_S7
##    57
```

Besides these two template datasets, users can also provide their pilot data for parameter estimation. If you want to use your dataset, it should be either a SingleCellExperiment object or a count matrix.

Scenario 1: Within cell type

For this scenario, we will use the template mouse data. Here we follow the POWSC vignette and focus on fibroblast cells.

Step 1-1: Parameter estimation

Users need to provide the template dataset or their pilot dataset and then call the Est2Phase function for parameter estimation.

```
# load template data(used for parameter estimation)
data('es_mef_sce')

# explore the data
table(es_mef_sce@colData$cellTypes)  # check the cell types

##
##    fibro stemCell
##       44       48

# subset the fibro cells
FIBRO <- es_mef_sce[,colData(es_mef_sce)$cellTypes=='fibro']

# if intereseted in stem cells:
# STEM <- es_mef_sce[,colData(es_mef_sce)$cellTypes=='stemCell']

set.seed(12)  # to ensure reproducible results
INDEX <- sample(1:nrow(FIBRO),600)  # the original data contains > 10000 genes. For illustrati
on purpose, we randomly select 600 genes.
FIBRO <- FIBRO[INDEX,]
est_paras <- Est2Phase(FIBRO)
str(est_paras)

## List of 7
##  $ exprs : num [1:600, 1:44] 65 0 78 233 121 0 0 0 35 29 ...
##   ..- attr(*, "dimnames")=List of 2
##   .. ..$ : chr [1:600] "Ankrd12" "Epas1" "Dnajc7" "r_MT2B" ...
##   .. ..$ : chr [1:44] "fibro_1" "fibro_2" "fibro_3" "fibro_4" ...
##  $ pi.g  : Named num [1:600] 0.705 0.177 0.563 0.811 0.68 ...
##   ..- attr(*, "names")= chr [1:600] "Ankrd12" "Epas1" "Dnajc7" "r_MT2B" ...
##  $ p0    : Named num [1:44] 0.852 0.866 0.815 0.927 0.947 ...
##   ..- attr(*, "names")= chr [1:44] "fibro_1" "fibro_2" "fibro_3" "fibro_4" ...
##  $ lambda: Named num [1:44] 1.54 2.25 2.51 3.56 1.46 ...
```

```
##   ..- attr(*, "names")= chr [1:44] "fibro_1" "fibro_2" "fibro_3" "fibro_4" ...
##  $ mu    : num [1:600] 11.2 10.4 10.7 10.8 11.5 ...
##  $ sd    : Named num [1:600] 1.35 1.36 1.5 1.76 1.5 ...
##   ..- attr(*, "names")= chr [1:600] "Ankrd12" "Epas1" "Dnajc7" "r_MT2B" ...
##  $ sf    : Named num [1:44] 0.06124 0.01029 0.07039 0.0312 0.0039 ...
##   ..- attr(*, "names")= chr [1:44] "fibro_1" "fibro_2" "fibro_3" "fibro_4" ...

est_paras2 <- Est2Phase(FIBRO,low.prob = 0.1)
```

The results are provided as seven lists: exprs, pi.g, p0, lambda, mu, sd and sf. exprs is the count matrix, and the rest correspond to π_g(length g), p_i, λ_i, μ_g and σ_g, respectively.

Step 1-2: Data simulation

After estimating the key parameters, we are ready to simulate scRNA-seq data. The function to call is Simulate2SCE.

```
simData <- Simulate2SCE(n = 200, perDE = 0.05, estParas1 = est_paras, estParas2 = est_para
s)
```

Four arguments are involved in this function, n is the total number of cells for two conditions; perDE is the percentage of DE genes, estParas1 and estParas2 are the set of parameters corresponding to cell type under two conditions, respectively. In this example, we simply adopt the same set of parameters. If users want to supply a different set of parameters for the two conditions, they need to make sure that the parameters' dimensions must be the same. i.e., estParas1 and estParas2 are obtained from two datasets of the same dimension (same number of genes and same number of cells).

The output from this function is a list of metrics, including the DE gene indices from Form I and II DE genes and simulated expression data (stored as SingleCellExperiment object).

```
## check the simulated data
simData$sce@assays@data$counts[1:4,1:4]

##    [,1] [,2] [,3] [,4]
## g1  116  196   46   35
## g2    0  123    0    0
## g3    0    0    0    0
## g4  449  520    0    0
```

Step 1-3: Power analysis

Next, we are ready to evaluate the power based on simulated data. Users can first call runDE function to identify DE genes, where they can choose either ‘MAST’ or ‘SC2P’ for DE_method. Then they call either Power_Cont (for the continuous case, i.e., Phase 2 DE genes) or Power_Disc (for the discrete case, corresponding to Phase 1 DE genes) for power evaluation.

```r
# identify DE genes
# user may also use either 'runMAST' or 'runSC2P'to call DE genes, which are just the same as runDE
DE <- runDE(simData$sce, DE_Method = 'MAST')  # another option for DE_method is 'SC2P'

## `fData` has no primerid.  I'll make something up.

## `cData` has no wellKey.  I'll make something up.

## Assuming data assay in position 1, with name et is log-transformed.

## (4.37,7.31] (7.31,8.27] (8.27,9.33] (9.33,10.5]   (10.5,15]
##     5.69766     5.69766     5.69766     6.55216     6.55216

##
## Done!

## Refitting on reduced model...

##
## Done!

# power evaluation, with FDR = 0.1
estPower1 <- Power_Cont(DE,simData, alpha = 0.1, delta = 0.3, strata = c(0,10,20,30,Inf))  # phase II DE
estPower2 <- Power_Disc(DE, simData, alpha = 0.1, delta = 0.1, strata = c(0,0.1,0.2,0.4,0.6,0.8,1))  # phase I  DE
#
```

In the Power_Cont function, users can freely modify ‘alpha’, ‘delta’ and ‘strata’ arguments. Specifically, alpha is the cutoff for the FDR, delta is the effect size, and in this case, it refers to log fold change. Strata specify how we will stratify the expression levels to report power.

In the Power_Disc function, the arguments are the same as those in Power_Cont, yet the meanings are slightly different. Specifically, delta is the cutoff for zero ratio change, and strata specify how we will stratify the zero proportions when reporting power.

The output lists power-related metrics, including marginal and stratified power.

```r
## Phase II DE genes
# marginal power
estPower1$power.marginal

## [1] 0.3076923

# stratified power
estPower1$power
```

```
## [1] 0.1250000 0.0000000 0.0000000 0.4666667

## Phase I DE genes
# marginal power
estPower2$power.marginal

## [1] 0.5789474

#stratified power
estPower2$power

## [1]       NaN 1.0000000 0.0000000 0.5000000 0.8571429 0.0000000
```

POWSC provides a single function named runPOWSC that wraps data simulation, DE identification, and power evaluation steps. Moreover, this function allows users to specify multiple sample sizes, facilitating easy comparison of power under a different number of cells. Besides, the output object can be used for plotting (the plot function in POWSC does not accept output from Power_Disc or Power_Cont).

A demonstration of runPOWSC is provided as follows:

```
sim_size <- c(100,200,300)
pow_rst <- runPOWSC(sim_size = sim_size,
                    per_DE = 0.05,
                    est_Paras = est_paras,
                    DE_Method = 'MAST',
                    Cell_Type = 'PW',
                    multi_Prob = NULL,
                    alpha = 0.1,
                    disc_delta = 0.1,
                    cont_delta = 0.3
                    )

## `fData` has no primerid.  I'll make something up.

## `cData` has no wellKey.  I'll make something up.

## Assuming data assay in position 1, with name et is log-transformed.

## (4.17,7.09] (7.09,8.05] (8.05,9.12] (9.12,10.3] (10.3,14.8]
##    5.274808    5.274808    5.274808    5.274808    6.920211

##
## Done!

## Refitting on reduced model...
```

```
##
## Done!

## `fData` has no primerid.  I'll make something up.

## `cData` has no wellKey.  I'll make something up.

## Assuming data assay in position 1, with name et is log-transformed.

## (4.24,7.16] (7.16,8.12] (8.12,9.19] (9.19,10.4] (10.4,14.9]
##    1.441090    5.613279    5.613279    5.650234    5.721656

##
## Done!

## Refitting on reduced model...

##
## Done!

## `fData` has no primerid.  I'll make something up.

## `cData` has no wellKey.  I'll make something up.

## Assuming data assay in position 1, with name et is log-transformed.

## (4.63,6.67]  (6.67,7.5]  (7.5,8.43] (8.43,9.45] (9.45,10.6] (10.6,14.8]
##    6.188738    6.188738   14.462959   14.462959   14.462959   14.462959

##
## Done!

## Refitting on reduced model...

##
## Done!
```

Most arguments have the same meaning as those in the separate functions for the data simulation, DE identification, and power evaluation. Some specific arguments are Cell_Type: specifying whether conduct within cell type DE identification (‘PW’) or between cell types DE identification (‘Multi’) multi_Prob: cell type proportions. Needs to sum up to 1. This argument is useful when conducting between-cell type DE identification. disc_delta: cutoff for zero ratio change. cont_delta: cutoff for the log fold change.

The results can be plotted using plot_POWSC.

```
plot_POWSC(pow_rst,Form = 'II',Cell_Type = 'PW')  # for phase II DEGs
```

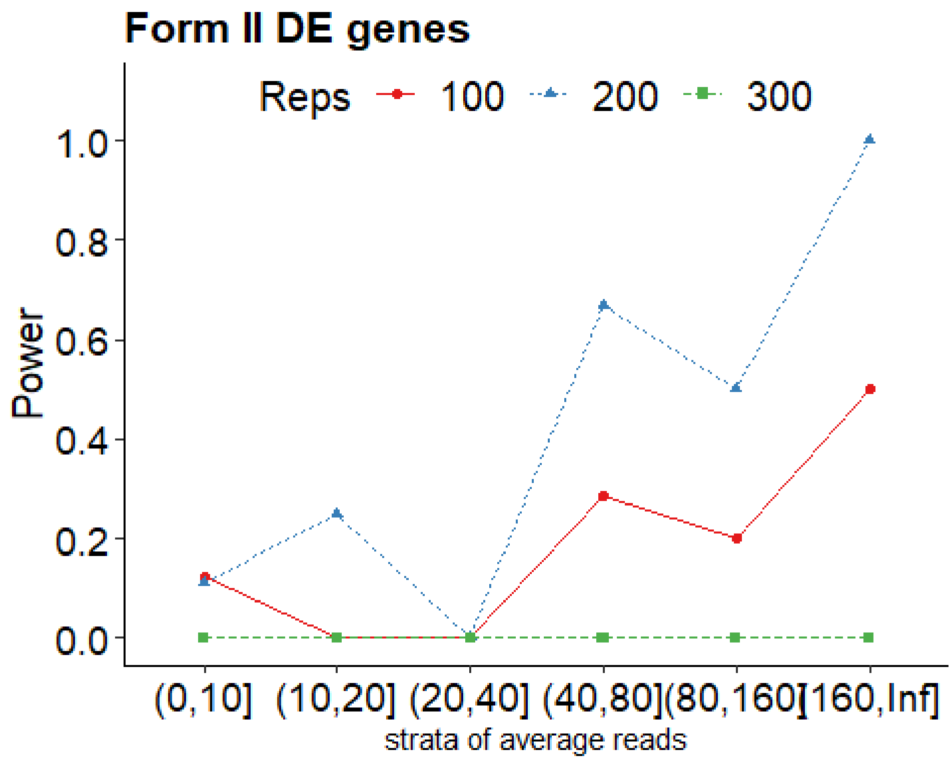


```
plot_POWSC(pow_rst,Form = 'I',Cell_Type = 'PW')
```

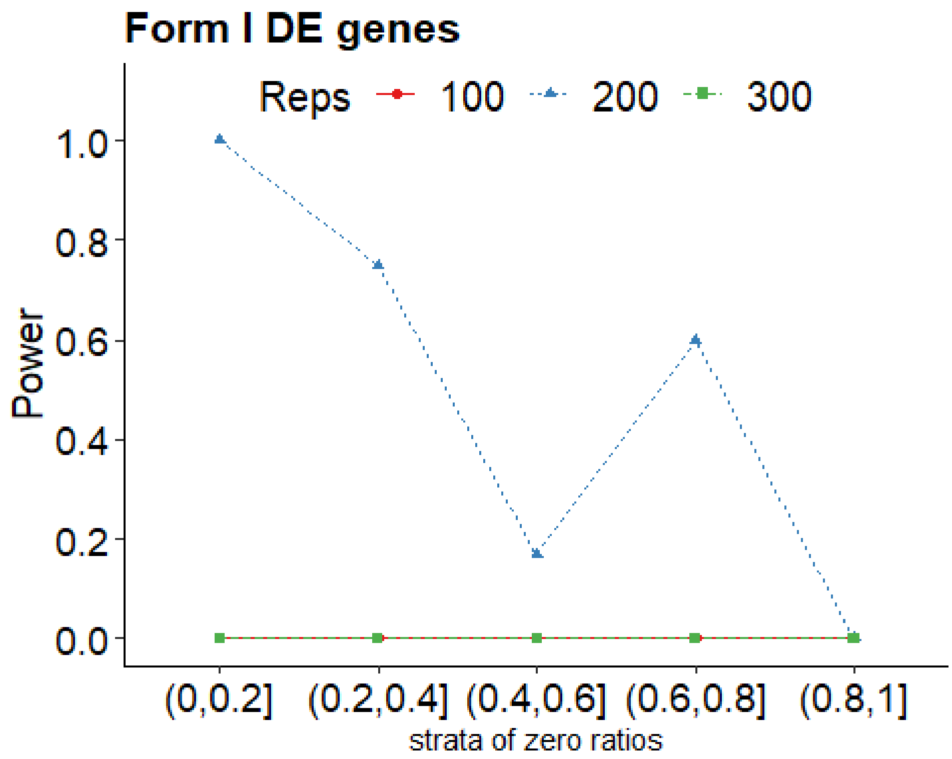

Or we can summarize the results by calling summary_POWSC function. The Form argument allows users to specify whether they want to show results for phase I DEGs or phase II DEGs.

```
summary_POWSC(pow_rst, Form= 'I',Cell_Type = 'PW')  # phase I DEGs

##     (0,0.2] (0.2,0.4] (0.4,0.6] (0.6,0.8] (0.8,1]
## 100       0      0.00    0.0000       0.0       0
## 200       1      0.75    0.1667       0.6       0
## 300       0      0.00    0.0000       0.0       0

summary_POWSC(pow_rst,Form = 'II', Cell_Type = 'PW') # phase II DEGs

##     (0,10] (10,20] (20,40] (40,80] (80,160] (160,Inf]
## 100 0.1250    0.00       0  0.2857      0.2       0.5
## 200 0.1111    0.25       0  0.6667      0.5       1.0
## 300 0.0000    0.00       0  0.0000      0.0       0.0
```

Scenario 2: Between cell types

In this scenario, the interest is to identify DEGs expressed differently between cell types. For demonstration, we use the human brain data (GSE67835). The template data in POWSC contains 57 cells, spanning four cell types, and all the cells come from the same patient. Here, we utilize the most abundant three cell types, namely hybrid, neurons, and oligodendrocytes. A series of datasets will be simulated, with the underlying cell type proportion being 20% (hybrid), 30% (oligodendrocytes), 50% (neurons), respectively. For each dataset, we will perform pairwise comparisons and report the power evaluation for each comparison.

Step 2-1: Parameter estimation

```
# load human brain data
sce

## class: SingleCellExperiment
## dim: 1000 57
## metadata(0):
## assays(1): counts
## rownames(1000): STIP1 STS ... LINC00311 PNKP
## rowData names(1): geneNames
## colnames(57): GSM1658127 GSM1658128 ... GSM1658182 GSM1658183
## colData names(3): tissueTypes cellTypes Patients
## reducedDimNames(0):
## mainExpName: NULL
## altExpNames(0):

# get expression data
exprs <- assays(sce)$counts  # 1000*57 count matrix
```

```
# estimate parameters for each cell types
col = colData(sce)
estParas_set = NULL
celltypes = c( "hybrid","neurons","oligodendrocytes")  #
for (cp in celltypes){
   print(cp)
   ix = grep(cp, col$cellTypes)
   tmp_mat = exprs[, ix]
   tmp_paras = Est2Phase(tmp_mat)
   estParas_set[[cp]] = tmp_paras
}

## [1] "hybrid"
## [1] "neurons"
## [1] "oligodendrocytes"
```

Step 2-2: Data simulation

For data simulation, users need to call SimulateMultiSCEs this time. There are four arguments in this function, where n specifies the number of total cells for multiple cell types; estParas_set : a set of estimated parameters for each cell type; multiProb: a vector of cell type proportions. No need to sum up to 1 (POWSC will normalize); delta1: the minimum of expression change used to determine the Form I DE; delta2: the minimum of log fold change used to determine the Form II DE.

```
sim_size <- 1000 # the number of
cell_per <- c(0.2,0.3,0.5)  # cell type proportions
sim <- SimulateMultiSCEs( n = sim_size,
                          estParas_set = estParas_set,
                          multiProb = cell_per,
                          delta1 = 0.1,
                          delta2 = 0.5)
```

The result is a list of the simulated dataset. Each dataset corresponds to a pairwise comparison, including indices from Form I and II DE genes and simulated expression data (stored as SingleCellExperiment object).

Step 2-3: Power analysis

For power analysis, we first identify DEGs using runDE function. Then call Power_Disc and Power_Cont functions to do power analysis for Form I and II DEGs, respectively. The usage of these functions is the same as before.

```
#### DE analysis
DE_rslt = NULL
for (comp in names(sim)){
```

```
    tmp = runDE(sim[[comp]]$sce, DE_Method = "MAST")
    DE_rslt[[comp]] = tmp
}

## `fData` has no primerid.  I'll make something up.

## `cData` has no wellKey.  I'll make something up.

## Assuming data assay in position 1, with name et is log-transformed.

## (4.17,4.84] (4.84,5.59] (5.59,6.44] (6.44,8.48]  (8.48,9.7]  (9.7,16.4]
##    6.289383    6.289383    6.289383    6.289383    6.289383    6.289383

##
## Done!

## Refitting on reduced model...

##
## Done!

## `fData` has no primerid.  I'll make something up.

## `cData` has no wellKey.  I'll make something up.

## Assuming data assay in position 1, with name et is log-transformed.

## (3.43,4.06] (4.06,4.78]  (4.78,5.6]   (5.6,7.6]  (7.6,8.83] (8.83,10.2]
##   0.9581862   0.9782294   7.2254917   7.2254917   7.2254917   7.2254917
## (10.2,15.7]
##   7.2254917

##
## Done!

## Refitting on reduced model...

##
## Done!

## `fData` has no primerid.  I'll make something up.

## `cData` has no wellKey.  I'll make something up.

## Assuming data assay in position 1, with name et is log-transformed.

## (3.79,4.44] (4.44,5.18] (5.18,6.02] (6.02,8.07]  (8.07,9.3]  (9.3,10.7]
##    4.873362    4.873362    7.218169    7.218169    7.218169    7.218169
## (10.7,16.2]
##    7.218169
```

```
##
## Done!

## Refitting on reduced model...

##
## Done!

#########
######### Summarize the power result
#########
pow_rslt = pow1 = pow2 = pow1_marg = pow2_marg = NULL
TD = CD = NULL
for (comp in names(sim)){
   tmp1 = Power_Disc(DE_rslt[[comp]], sim[[comp]])
   tmp2 = Power_Cont(DE_rslt[[comp]], sim[[comp]])
   TD = c(TD, tmp2$TD); CD = c(CD, tmp2$CD)
   pow1_marg = c(pow1_marg, tmp1$power.marginal)
   pow2_marg = c(pow2_marg, tmp2$power.marginal)
   pow_rslt[[comp]] = list(pow1 = tmp1, pow2 = tmp2)
   pow1 = rbind(pow1, tmp1$power)
   pow2 = rbind(pow2, tmp2$power)
}

######### Demonstrate the result by heatmap
#########
library(RColorBrewer); library(pheatmap)

## Warning: package 'RColorBrewer' was built under R version 4.1.3

breaksList = seq(0, 1, by = 0.01)
colors = colorRampPalette(rev(brewer.pal(n = 7, name = "RdYlBu")))(length(breaksList))
dimnames(pow1) = list(names(sim), names(tmp1$CD))
dimnames(pow2) = list(names(sim), names(tmp2$CD))

## visualize the results for Form I DEGs
pheatmap(pow1, display_numbers = TRUE, color=colors, show_rownames = TRUE,
        cellwidth = 30, cellheight = 40, legend = TRUE,
        border_color = "grey96", na_col = "grey",
        cluster_row = FALSE, cluster_cols = FALSE,
        breaks = seq(0, 1, 0.01),
        main = "")
```

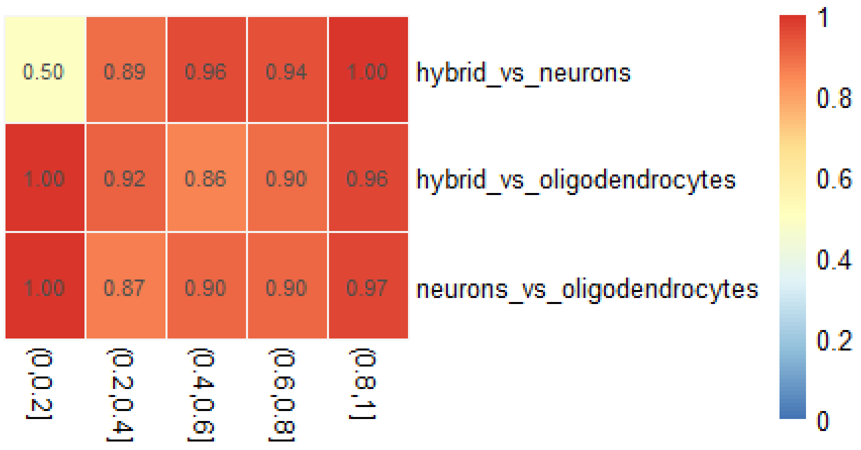


```
## visualize the results for Form II DEGs
pheatmap(pow2, display_numbers = TRUE, color=colors, show_rownames = TRUE,
         cellwidth = 30, cellheight = 40, legend = TRUE,
         border_color = "grey96", na_col = "grey",
         cluster_row = FALSE, cluster_cols = FALSE,
         breaks = seq(0, 1, 0.01),
         main = "")
```

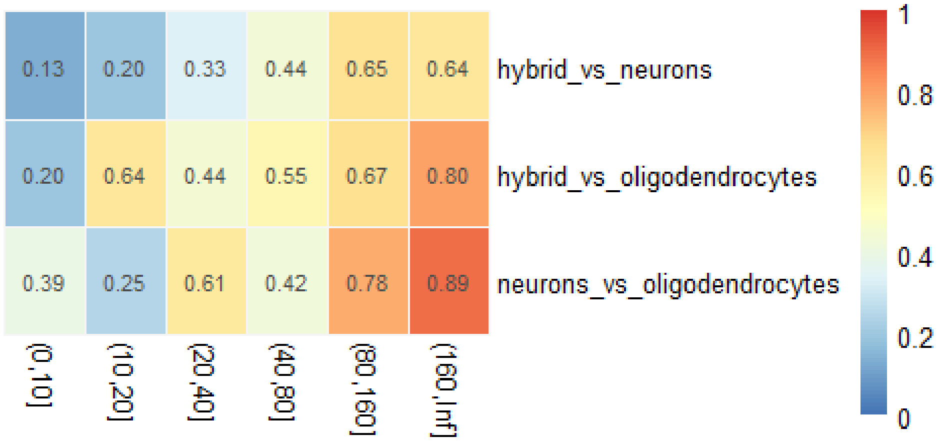

And like the case for within cell type, we can use runPOWSC, which wraps simulation, DE analysis, and power evaluation, and also allows for comparing different sample sizes.

```
powsc_rst <- runPOWSC(
    sim_size = c( 200, 800, 1000),
    per_DE = 0.05,
    est_Paras = estParas_set,
    DE_Method = "MAST",
    Cell_Type = "Multi",
    multi_Prob = cell_per,
    alpha = 0.1,
    disc_delta = 0.1,
    cont_delta = 0.5
)

## `fData` has no primerid.  I'll make something up.

## `cData` has no wellKey.  I'll make something up.

## Assuming data assay in position 1, with name et is log-transformed.

## (3.53,4.92] (4.92,5.77] (5.77,6.75] (6.75,7.86] (7.86,9.13] (9.13,10.6]
##    4.342940    4.342940    6.327037    6.327037    6.327037    6.327037
```

```
## (10.6,16.3]
##    6.663939

##
## Done!

## Refitting on reduced model...

##
## Done!

## `fData` has no primerid.  I'll make something up.

## `cData` has no wellKey.  I'll make something up.

## Assuming data assay in position 1, with name et is log-transformed.

## (2.99,4.32] (4.32,5.15] (5.15,7.21] (7.21,8.49] (8.49,9.97] (9.97,15.9]
##    1.195028    5.412472    5.412472    5.412472    5.412472    6.339718

##
## Done!

## Refitting on reduced model...

##
## Done!

## `fData` has no primerid.  I'll make something up.

## `cData` has no wellKey.  I'll make something up.

## Assuming data assay in position 1, with name et is log-transformed.

## (3.31,4.69] (4.69,5.53] (5.53,7.61] (7.61,8.88] (8.88,10.3] (10.3,16.2]
##    7.041537    7.041537    7.041537    7.041537    7.041537    7.041537

##
## Done!

## Refitting on reduced model...

##
## Done!

## `fData` has no primerid.  I'll make something up.

## `cData` has no wellKey.  I'll make something up.

## Assuming data assay in position 1, with name et is log-transformed.
```

```
##  (4.23,4.9]  (4.9,5.66] (5.66,7.48] (7.48,8.56] (8.56,9.79] (9.79,16.5]
##    4.601535    4.601535    4.601535    4.601535    4.601535    4.601535

##
## Done!

## Refitting on reduced model...

##
## Done!

## `fData` has no primerid.  I'll make something up.

## `cData` has no wellKey.  I'll make something up.

## Assuming data assay in position 1, with name et is log-transformed.

##  (3.5,4.13] (4.13,4.85] (4.85,5.67] (5.67,7.68]  (7.68,8.9]  (8.9,10.3]
##    1.154594    1.154594    6.162667    6.162667    6.162667    6.162667
## (10.3,15.8]
##    6.162667

##
## Done!

## Refitting on reduced model...

##
## Done!

## `fData` has no primerid.  I'll make something up.

## `cData` has no wellKey.  I'll make something up.

## Assuming data assay in position 1, with name et is log-transformed.

##  (3.8,4.45] (4.45,5.19] (5.19,6.99] (6.99,8.07]  (8.07,9.3]  (9.3,10.7]
##    7.267414    7.267414    7.267414    7.267414    7.267414    7.267414
## (10.7,16.1]
##    7.267414

##
## Done!

## Refitting on reduced model...

##
## Done!

## `fData` has no primerid.  I'll make something up.
```

```
## `cDataˋ has no wellKey.  I'll make something up.

## Assuming data assay in position 1, with name et is log-transformed.

## (4.28,4.95] (4.95,5.71] (5.71,7.52]  (7.52,8.6]  (8.6,9.81] (9.81,16.4]
##    6.383678    6.383678    6.383678    6.383678    6.383678    6.410608

##
## Done!

## Refitting on reduced model...

##
## Done!

## `fData` has no primerid.  I'll make something up.

## `cData` has no wellKey.  I'll make something up.

## Assuming data assay in position 1, with name et is log-transformed.

## (3.29,3.92] (3.92,4.63] (4.63,5.46] (5.46,7.47] (7.47,8.71] (8.71,10.1]
##    7.240856    7.240856    7.240856    7.240856    7.240856    7.240856
## (10.1,15.7]
##    7.240856

##
## Done!

## Refitting on reduced model...

##
## Done!

## `fData` has no primerid.  I'll make something up.

## `cData` has no wellKey.  I'll make something up.

## Assuming data assay in position 1, with name et is log-transformed.

## (3.71,4.36]  (4.36,5.1]  (5.1,5.94] (5.94,7.98] (7.98,9.22] (9.22,10.6]
##    6.280485    6.280485    6.280485    6.280485    6.280485    6.280485
## (10.6,16.1]
##    6.280485

##
## Done!

## Refitting on reduced model...
```

```
##
## Done!

## results visualization
plot_POWSC(powsc_rst, Form = 'I', Cell_Type = 'Multi') # Form I DEGs
```

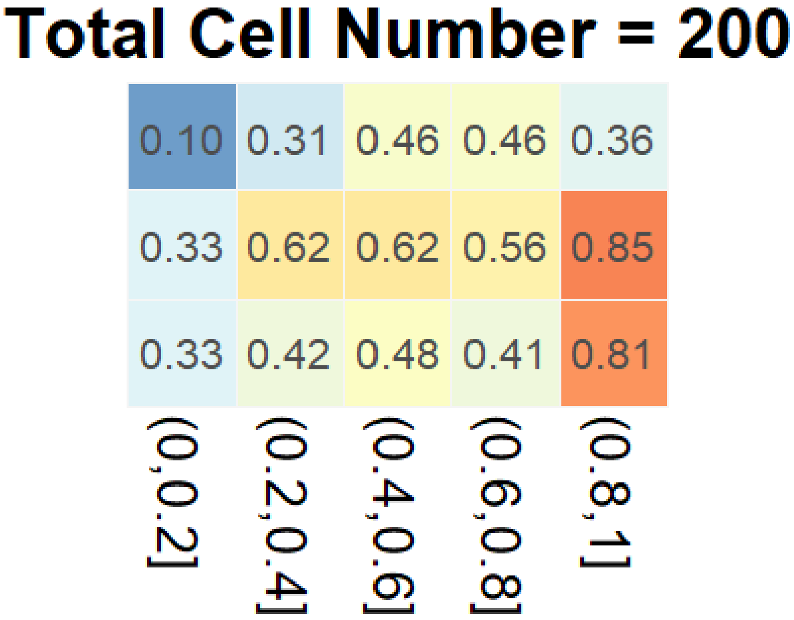
Total Cell Number = 200
0.10 0.31 0.46 0.46 0.36
0.33 0.62 0.62 0.56 0.85
0.33 0.42 0.48 0.41 0.81
(0,0.2]
(0.2,0.4]
(0.4,0.6]
(0.6,0.8]
(0.8,1]

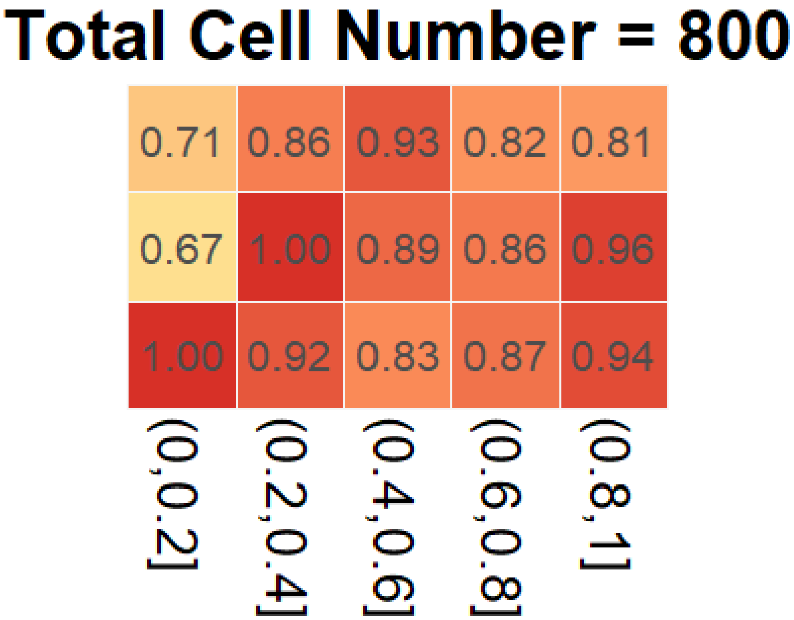
Total Cell Number = 800
0.71 0.86 0.93 0.82 0.81
0.67 1.00 0.89 0.86 0.96
1.00 0.92 0.83 0.87 0.94
(0,0.2]
(0.2,0.4]
(0.4,0.6]
(0.6,0.8]
(0.8,1]

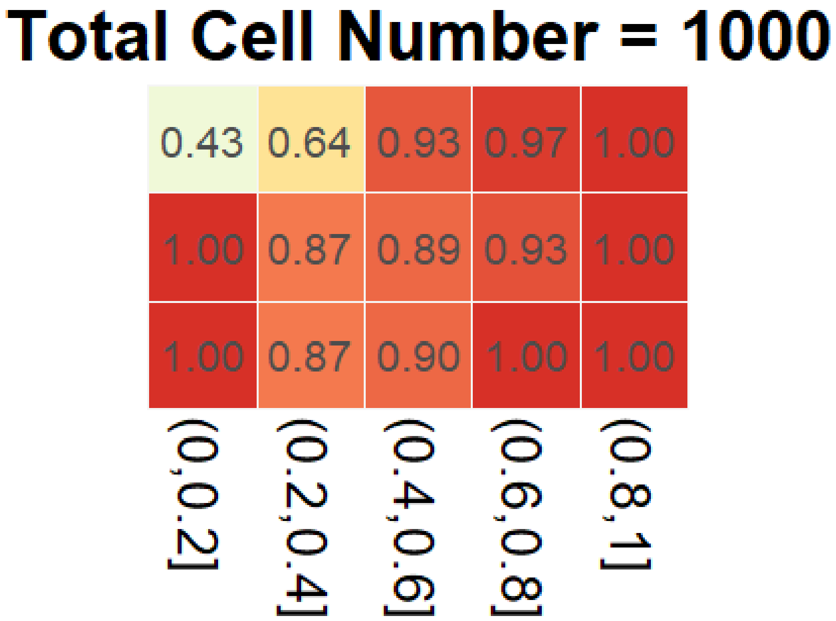


```r
plot_POWSC(powsc_rst,Form = 'II',Cell_Type = 'Multi')  # Form II DEGs
```

Total Cell Number = 200

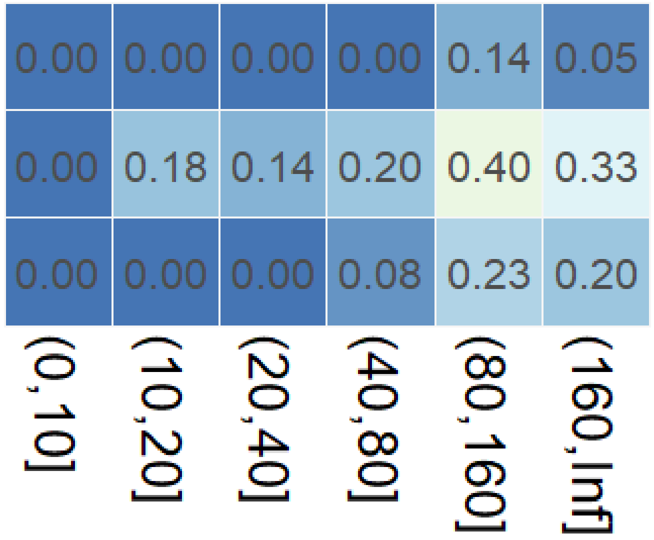

Total Cell Number = 800

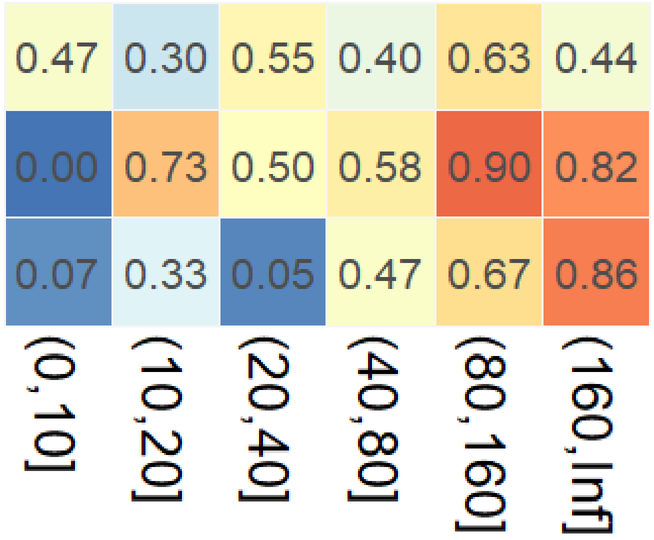

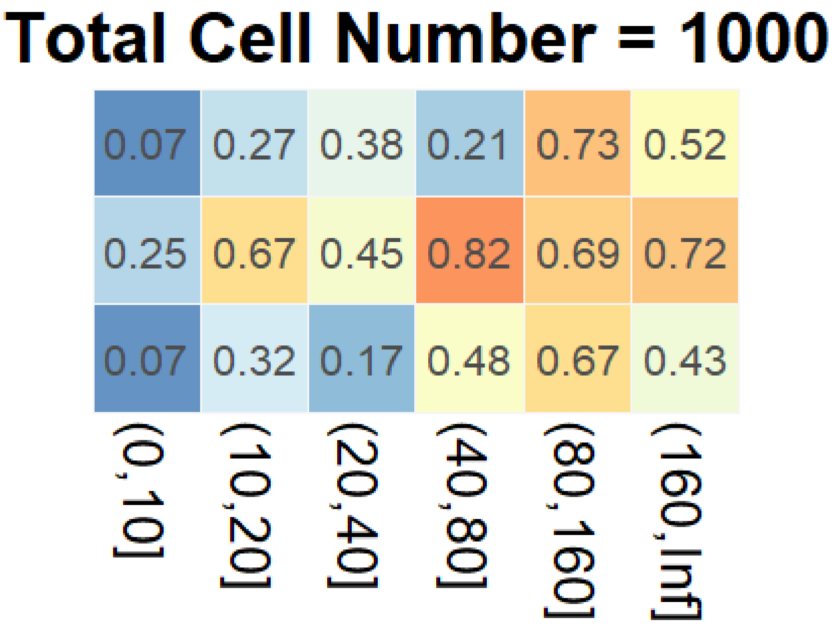
Total Cell Number = 1000
0.07 0.27 0.38 0.21 0.73 0.52
0.25 0.67 0.45 0.82 0.69 0.72
0.07 0.32 0.17 0.48 0.67 0.43
[0,10]
(10,20]
(20,40]
(40,80]
(80,160]
(160,Inf]
